# Importance of Shared Decision-Making for Vulnerable Populations: Examples from Postmastectomy Breast Reconstruction

**DOI:** 10.1089/heq.2018.0020

**Published:** 2018-09-01

**Authors:** Victoria F. Grabinski, Terence M. Myckatyn, Clara N. Lee, Sydney E. Philpott-Streiff, Mary C. Politi

**Affiliations:** ^1^ Division of Public Health Sciences, Department of Surgery, Washington University School of Medicine, St. Louis, Missouri.; ^2^ Division of Plastic and Reconstructive Surgery, Department of Surgery, Washington University School of Medicine, St. Louis, Missouri.; ^3^ Department of Plastic and Reconstructive Surgery, College of Medicine, The Ohio State University, Columbus, Ohio.; ^4^ Division of Health Services Management and Policy, College of Public Health, The Ohio State University, Columbus, Ohio.

**Keywords:** patient–provider communication, shared decision-making, vulnerable populations

## Abstract

Shared decision-making (SDM) is a process through which patients and providers collaborate to select a treatment option that aligns with patients' preferences and clinical context. SDM can improve patients' decision quality and satisfaction. However, vulnerable populations face barriers to participation in SDM, which exacerbates disparities in decision quality. This perspective article discusses SDM with vulnerable patients, using examples from patients who made decisions about postmastectomy breast reconstruction. We offer several strategies for clinical practice, medical education, and research to ensure that movements to engage patients in SDM do not exclude already marginalized groups.

## Introduction

I thought [only] people in Hollywood had reconstructed breasts. I was a poor, old, Black lady, and it didn't matter whether I had one breast… I gladly would have talked with somebody if somebody had approached me with the details about it… I guess they felt like a woman my age, you don't matter. You're gettin’ ready to die anyway. You may as well die with one breast.

These unfortunate comments were made by one breast cancer survivor recounting her experiences with postmastectomy breast reconstruction in our recent study about shared decision-making (SDM).^1^ No patients should be left feeling that they received suboptimal care or were treated differently because of their race, age, or socioeconomic status (SES). This patient felt that her treatment needs were not met because her healthcare team made inaccurate assumptions about her preferences based on preconceived beliefs. She later recounted that the treatment team did not adequately address her questions about breast reconstruction access or cost concerns.

SDM is defined by patient–provider collaboration in preference-sensitive treatment decisions.^2^ SDM helps patients choose a treatment that is aligned with both their preferences and clinical context.^3^ This crucial practice helps increase patient satisfaction with their healthcare. It is particularly well suited for treatment decisions that are elective and/or preference sensitive such as the postmastectomy breast reconstruction example mentioned earlier. Patients considering breast reconstruction must decide whether to have the procedure, when to have it done (immediate or delayed), and what type to have (implant or autologous, which uses the patient's own tissue).^1^ Each option presents distinct trade-offs that patients must weigh. For example, breast reconstruction can restore body image and improve quality of life.^4^ However, the risk of complications such as infection or tissue necrosis is approximately 23% 2 years after immediate reconstruction.^5–8^ Delaying reconstruction may lower the risk of complications.^9^ Evidence and guidelines suggest that SDM should be encouraged in these types of clinical situations,^10^ and providers generally support the broad idea of engaging patients through SDM.^11^ Yet, some patient populations continue to report barriers to engaging with providers about preference-sensitive decisions.^12,13^ In this context, we define vulnerable patients as those who are economically disadvantaged, racial and ethnic minorities, uninsured, low income, and/or elderly.^14,15^ Patients with multiple vulnerabilities may benefit from additional support to meaningfully engage with providers.

This perspective article explores disparities in the quality of SDM based on the literature, clinical experience, and a theme that emerged from our recent study about postmastectomy breast reconstruction decision-making. The methodology of that IRB-approved qualitative study is described in detail elsewhere.^1^ In this perspective article, we highlight barriers to SDM experienced by vulnerable patient populations using illustrative quotes to support these statements when appropriate. We offer some strategies for future research and clinical training to ensure that movements to engage patients in SDM do not exclude already marginalized groups.

## Barriers to SDM for Vulnerable Populations

Although barriers to engaging in SDM are not unique to vulnerable groups,^11,16^ SDM with vulnerable populations requires additional considerations. For example, some patients may distrust the healthcare system or experience heightened power differentials with their providers; distrust and power imbalances presents challenges for partnership through SDM.^12^ Implicit biases held by providers can also limit patients' options and exclude them from the decision-making process, as this patient quote from our recent study demonstrates^1^:

He [surgeon] said, ‘Well, you're old anyhow, so what difference does it make?’ Isn't that cruel? Those were the words. My husband and I were just in shock… I just cried for the rest of the day.

Biases can also inhibit information sharing with vulnerable patients. For example, doctors are less likely to support their recommendations with research findings and clinical experience when counseling minority patients.^17^ This inequitable communication can exacerbate disparities in the quality of patient decision-making.^18^ Patients may feel that the information they receive is not representative or applicable to them. As a patient in our study commented:

I saw very little people—women of color that had had implants or reconstruction all together. Those photos were not available… Representation really matters… It really matters to have people that look like you… and normalize that experience for each individual goin' through it…

A related barrier to SDM is the ability of the patient to confidently communicate with their provider about options and preferences. Nonwhite and older individuals are more likely to have low health literacy,^19^ which limits their ability to engage in SDM. Supplementing conversations with decision tools can help address information gaps, but tools to support SDM are often not developed with specific attention to the needs of vulnerable populations.^20^ In addition, educational materials may not communicate information in ways that facilitate comprehension, such as using icon arrays to present risk.^21^ One patient in our study was left with an incomplete understanding of surgical risks after reading complicated educational materials.

Yes, I read it [the pamphlet], but that doesn't mean I quite understood it.

Similarly, many educational tools in the United States are written exclusively in English, without available translations in other languages. They may have been developed without considering cultural norms of nonwhite and non-English-speaking populations. In addition, older patients may prefer to use paper-based rather than electronic tools, yet this format is not always available. Physicians may misinterpret communicative style among some patients with lower SES as less willingness to be involved in the decision-making process.

## Clinical Practice and Research Implications

We recommend several strategies for successful implementation of SDM with vulnerable populations (Table 1). In addition to providers' commitment to engaging patients, several practical interventions can bolster SDM for these individuals. First, providers should be trained in SDM and how it can be used in clinical practice. Those who are familiar with SDM may be wary of the additional time that it requires.^11,16^ However, evidence suggests that the average time required for SDM is <3 min,^22^ and it can be accomplished efficiently during consultations with practice. Training during medical education may help providers develop specific SDM skills and feel comfortable with the concept of greater patient participation.

**Table 1.** Summarizing Support Strategies for Engaging in Shared Decision-Making with Vulnerable Populations

Barrier identified	Suggestion for clinical practice	Sample clinician wording
Lack of provider training in SDM can further marginalize vulnerable patients	Increased SDM training in medical education and renewed commitment to SDM implementation with vulnerable patients	“There are several options to consider. I use this decision tool to help you learn about some of these options. We can then talk about the options and how you feel about them in more detail.”
Provider biases and assumptions can impact which options are offered to patients and how competing options are presented	Deliberate perspective taking, implicit bias training for healthcare providers, probing patients' own values, and structuring conversations around the concerns they identify as personally relevant or critical to their decision	“Either procedure x or y is a good option in this case. The pros and cons of procedure x are these, and those of y are these. How do you feel about those options, now that you have some information about them?”
Lacking cost discussions across socioeconomic strata, which fails to address economic burden and limits access for patients with low SES	Discussing costs even if particular figures are not known, referral to social workers and financial case managers for more detailed information, improved training in cost communication during medical education, and increased price transparency	“When making your decision, you may want to think about insurance co-pays, time away from work, and having more clinic visits and surgeries this year. Insurance covers reconstruction, but some people want to discuss how much they might pay out-of-pocket. We can set you up with someone who can talk through your costs if you want to learn more.”
Limited applicability of decision aids for vulnerable patients; information may be unrepresentative for some groups and not accessible for those with low health literacy, widening knowledge gaps	Feedback from a diverse group of potential users during development, incorporation of the particular concerns that vulnerable patients face, representation or personalization of information, tailoring to low reading level, including pictorially presented information; implementing decision aids with attention to vulnerable patients' needs	“We also have a video you can watch and pictures you can look at. You can review everything at your own pace. We can meet again before your surgery to make sure you have a clear understanding of your options, or I can set up a time to call you.”
Availability of interventions and poor reimbursement may limit SDM	Legislation and policy initiatives to promote the adoption of SDM, including incorporation into reimbursement structures and SDM mandates for preference-sensitive procedures	[Clinician wording is not applicable to this system-level intervention.]

SDM, shared decision-making.

In addition, to thoughtfully engage with vulnerable patients, providers should consider unique challenges experienced by vulnerable groups. Chief among these are issues of cost, which affect patients' treatment preferences^23^ and disproportionately burden vulnerable groups. Yet, cost considerations are excluded from the vast majority of clinical encounters. One patient in our study reported dissatisfaction with her choice because she was not aware that the procedure was mandated by law to be covered by insurance.

When you talk about reconstruction that meant, oh, you're rich. I was never told otherwise.

When cost is discussed, most cost conversations last <1 min.^24^ Many patients feel too uncomfortable asking their provider about care costs.^25^ Although cost conversations can feel awkward for physicians as well, even giving patients comparative information about the costs of different options is helpful. For more detailed information, physicians could direct patients to other providers with more experience navigating cost conversations, such as social workers and financial case managers.^23^

To better inform patients and increase their self-efficacy communicating about treatment decisions, providers could direct patients to resources that provide accurate representative information at an accessible reading level. For example, pictorial representations of risk for patients with limited literacy or numeracy and paper formats for older patients can facilitate understanding.^21^ Resources and information in plain language that promote improved treatment knowledge can empower patients to engage in their decision through SDM. Providers should also encourage and normalize patient participation in treatment decision-making to avoid the decisional regret experienced by this older, economically disadvantaged black patient in our study:

I was stuck with a mastectomy and that's it. I would have liked to have known or [be] given information on reconstruction but that never happened.

Across the board, empowering patients to express their concerns and preferences promotes improved communication.

SDM has the potential to inform patients about their options and help them choose the treatment that is most appropriate for their unique needs. However, that treatment may not be preferred by providers because of concerns about their clinical practice. For example, autologous breast reconstruction with microsurgery may be the best option for a patient, particularly if she has had radiation or a failed implant-based reconstruction.^26^ However, it is commonly performed by only a minority of reconstructive surgeons. Thus, providers or healthcare systems that do not offer this technique may not want to use SDM, out of concern that patients will prefer this procedure. Unfortunately, financial reimbursement may also dissuade providers from adopting SDM tools in instances when poorly reimbursed treatments are more likely to be preferred. For example, autologous breast reconstruction is more resource intensive than implant-based reconstruction, yet is reimbursed less per hour.^27,28^ At times, SDM may need to be supported by legislation or policy incentives to ensure that patients, particularly those of limited socioeconomic means, receive the most appropriate care.^3,29^ Improved implementation at the institutional level may also be necessary to improve SDM accessibility.^30^

Vulnerable patients should also be included in research studies aimed at designing clinical decision aids to widen the accessibility and impact of these tools. Developers should consistently seek feedback from diverse patient populations to increase the applicability of their tools and to harness their potential to decrease knowledge gaps for all users. Efforts to implement SDM in clinical practice should be based on sound principles of implementation science and consider vulnerable patients as key stakeholders from the onset.^20^

## Conclusion

Although important for all patients, patients from vulnerable populations, including those with limited health literacy, low SES, and racial and ethnic minorities, can particularly benefit from a patient-centered SDM approach.^13,20^ Those with multiple vulnerable identities may benefit from additional support and resources to engage in SDM. Because of its potential to empower patients to participate in their care, SDM is especially important for those populations who may feel excluded from the medical decision-making process. To be successful at achieving its goals of preference-aligned, patient-centered care for all individuals, SDM requires inclusiveness in decision support materials, plain language communication, and attention to potential bias in clinical encounters. Thoughtful implementation of SDM can empower marginalized patients with more autonomy in their treatment and may reduce disparities in access to care and improved care outcomes.

## Notes

### Abbreviations Used

SDM: shared decision-making
SES: socioeconomic status
